# ADCY5 act as a putative tumor suppressor in glioblastoma: An integrated analysis

**DOI:** 10.1016/j.heliyon.2024.e37012

**Published:** 2024-08-27

**Authors:** Wang Can, Wen Yan, Huang Luo, Zhang Xin, Luo Yan, Liu Deqing, Tu Honglei, Li Xiaoyu, Sui Jiangdong, Xie Yue, Li Jing

**Affiliations:** a Chongqing Key Laboratory of Translational Research for Cancer Metastasis and Individualized Treatment, Chongqing University Cancer Hospital, Chongqing, 400030, China; b Department of Nephrology, The Ninth People's Hospital of Chongqing, Chongqing, 400700, China; c Department of Dermatology, Chongqing Traditional Chinese Medicine Hospital, Chongqing, 400011, China

**Keywords:** ADCY5, GBM, Proliferation, Migration, Bioinformatics analysis, Prognostic value

## Abstract

**Background:**

Adenylyl cyclase (AC) isoforms played a key role in the multiple cancer pathology, However, the expression, prognostic value and function of ADCY5 in Glioblastoma (GBM) have not been reported yet. This research intends to discover the expression, epigenetic alteration and biological function of ADCY5 in GBM and its value on patients' prognosis.

**Methods:**

① Transcriptional level, epigenetic alteration, prognostic value and molecular network of ADCY5 were analyzed by using of public online datasets. ② The mRNA expression profile of ADCY5 was explored by using GEPIA database and protein expression levels were detected by HPA Database. ③ The prognostic value of ADCY5 was determined by Kaplan-Meier Plotter, GEPIA and CGGA database. ④ The epigenetic characteristics of ADCY5 were determined by DiseaseMeth database. ⑤ Identification of genes co-expressed with ADCY5 and potential mechanism analyses were performed by using DAVID cBioPorta and STRING. ⑥ Reverse transcription-polymerase chain reaction (RT-PCR), cell counting kit-8 (CCK-8), colony formation, wound-healing scratch and transwell assay were applied to detect relative mRNA expression and biological function of ADCY5 in GMB cells.

**Results:**

ADCY5 mRNA and protein were downregulated in GBM compared with normal tissues. Analysis of the genetics and epigenetics of ADCY5 suggested that its expression was negatively correlated with DNA methylation. High expression of ADCY5 was significantly associated with age, grade, IDH mutation, 1p19q_codeletion, radiotherapy and chemotherapy and acted as an independent prognostic factor in GBM. ADCY5 mRNA also down-expressed in GBM cell lines and re-expressed of ADCY5 could inhibit cell proliferation, viability, migration/invasion and epithelial-mesenchymal transition (EMT) in vitro. In the analysis of genes co-expressed with ADCY5, we found that cAMP/AKT pathway, cGMP-PKG pathway, Wnts pathway were dissimilarly enriched.

**Conclusion:**

Our study indicated that ADCY5 could act as an epigenetic biomarker in GBM, as well as a prognosis target in patients with GBM.

## 1 Introduction

GBM is the most common and malignant tumor in the central nervous system (CNS), and with the mean survival time of GBM is approximately 15 months [1,2]. GBM originated from neuroectoderm, formed by glial or precursor cells and accounted for more than 80 % of primary malignant brain tumors [3]. The standard therapy for GBM mainly focused on maximizing the removal of tumor tissues to alleviate intracranial hypertension and neurological symptoms. Postoperative radiation therapy combined with temozolomide chemotherapy were standard treatment methods, but the effectiveness was limited. Currently, molecular targeted therapy shows promising results in various tumors. Therefore, it is crucial to explore new treatment methods for GBM. Thus understanding the pathogenesis of GBM at molecular levels is particularly important in identifying new therapeutic targets.

Adenylate cyclase 5 (ADCY5) is a member of ACs families, which converts adenosine triphosphate into the second messenger cyclic adenosine monophosphate (cAMP) and pyrophosphate [4,5]. cAMP could regulate numerous intracellular signaling pathways in various ways. Due to the different tissue distribution of ACs subtypes, cAMP originated from specific ACs could selectively and differentially regulated effector proteins. These subtypes usually integrated different cellular signals and acted as key enzymes to activate downstream signaling pathways [6,7].

Previous studies about ADCY5 mainly focused on energy metabolism, lipometabolism, blood sugar-related indicators, type 2 diabetes, and gestational diabetes [8]. Moreover, multiple studies had shown that ADCY5 may affect glucose metabolism through glucose coupled insulin secretion in human pancreatic islets [8,9]. Recently some researchers begun to pay attention to the effects of the ACs in carcinogenesis. For example, ADCY1 was responsible for catalyzing ATP to cyclic AMP (cAMP) and could influence platinum-based chemotherapy response in non-small cell lung cancer (NSCLC) [10]. ADCY7 was abnormally expressed in multiple human cancers and correlated with MMR genes and DNMT expression [11]. ADCY9 polymorphisms increased colorectal cancer risk in the Chinese Han population [12]. However, the expression levels, methylation, underlying function and prognostic significance of ADCY5 in GMB remained obscure. A combined study of ADCY5 in GBM is urgently needed.

## 2 Materials and methods

### 2.1 Cell culture and transfection

GBM cell lines (U87, LN-18, A172, U373, T98G, G28, G-267) and Human Astrocyte NHA were obtained from the Cell Bank of the Chinese Academy of Sciences (CBCAS, Shanghai, China), which would maintain in 10 % Fetal bovine serum (FBS) with 1 % (10 mg/ml) Penicillin Streptomycin double antibody RPMI-1640 medium (ThermoFisher). These above cells were maintained at an environment of 37 °C and 5 % CO2. pcDNA3.1(+)-ADCY5 and pcDNA3.1(+)-Vector were purchased from FuNeng GENE Company (Guangzhou, China). Each of them was transfected into cells by using Lipofectamine2000 (Life Technologies). After 48 h, the cells would be trypsinized and resuspended for further experiment.

### 2.2 CGGA database analysis

CGGA (Chinese Glioma Genome Atlas) database was comprised of clinical and sequencing data with over 2000 brain tumor samples from Chinese cohorts, which was equipped with a user-friendly web application for data storage and exploration. CGGA allowed for rapid assessment of gene expression levels, identification of co-expressed genes and had association with outcomes of single genes, gene sets or gene signatures. Moreover, CGGA offered the possibility of investigation of gene expression levels in Glioma subgroups, as well as analysis of clinical factors. ADCY5 mRNA expression were taken from mRNAseq_325, mRNAseq_693 and mRNAseq_array_301 dataset. The information of methylation was obtained from Methyl_159 dataset.

### 2.3 GEPIA and HPA database analysis

GEPIA2 is a web server for analyzing the RNA sequencing expression data of cancer and normal samples from the TCGA and the GTEx projects pipeline. GEPIA provided multiple cancer functions including Isoform-level Expression Analyses, Cancer Subtypes Analyses, Signature Score Analyses, Upload-data for Analysis. The GEPIA2 unique features Analysis, Isoform Structure Analysis, Cancer Subtype Classifier Analysis and Expression Analysis. The thresholds were restricted as follows: Log2FC/Cutoff = 1, P-value = 0.01, Jitter Size = 0.4 and Match TCGA normal and GTEx data.

HPA database(https://www.proteinatlas.org/) was initiated to map all human proteins in cells, tissues, and we explored ADCY5 protein expression levels in GBM and normal tissues by immunohistochemistry organs. Antibodies: HPA017730.

### 2.4 MethHC dataset analysis

The methylation status of ADCY5 in GBM was detected through MethHC dataset and DiseaseMeth analysis. In terms of the methylation of ADCY5 gene in GBM, different comparisons between GBM and normal tissues were analyzed as described before [13]. Promoter (from −1.5 to 0.5 kb of the transcription start site, TSS), TSS1500, TSS200, 5′UTR, first exon, gene body and 3′UTR gene region.

### 2.5 Reverse transcription (RT)-PCR and real-time PCR (qRT-PCR)

Total RNA was obtained from using Trizol reagent (Invitrogen). 2 μl separated RNA was reverse transcribed using the PrimeScript™ Reverse Transcription System with cDNA Eraser (TAKARA) based on the manufacturer's instructions. Semi-quantitative RT-PCR was carried out with Go-Taq DNA polymerase (Promega, Madison, WI, 487 USA) under the conditions detailed in a previous study [14,15]. qRT-PCR was carried out using SYBR® Green Master Mix (TAKARA) and measured through the 7500 Real-Time PCR System (ABI Prism 7500 FAST) as described before [14,15]. All PCR products were sequenced to confirm that correct products were obtained. The experiments were repeated three times and the primers were listed in Table 1, Table 2.

**Table 1:** List of RT-PCR primers used in this study.

Primer	Sequence (5′–3′)	Product size (bp)	PCR cycles
ADCY5-F	CAGAGAACTGGATCACGC	202	32
ADCY5-R	GGAATTTAAGGAGAGAAGGC		
MKI67-F	CAGACATCAGGAGAGACTACAC	242	32
MKI67-R	GTTAGACTTGCTGCTGAGTCTA		
E-cadherin-F	GGCATTGGGAAGAATCAGCC	287	32
E-cadherin-R	ATTGATGTGTCCAATGGCCG		
N-cadherin-F	CTATGAGTGGAACAGGAACGC	181	32
N-cadherin-R	TCTCGGCCTCTTGAGGTAAC		
Vimentin-F	TCGCCAACTACATCGACAAG	276	32
Vimentin-R	AAGATTGCAGGGTGTTTTCG		
GAPDH-F	GTGATGGGATTTCCATTGAT	206	23
GAPDH-R	GTGATGGGATTTCCATTGAT		

F, forward; R, reverse.

**Table 2:** List of qRT-PCR primers used in this study.

Primer	Sequence (5′–3′)	Product size (bp)	PCR cycles
AKT1-F	AATACCTGGTGTCGGTCTCA	152	40
AKT1-R	TCGAGCTCATCCTAATGGAG		
AKT2-F	ATGGTAGCCAACAGTCTGAAGC	136	40
AKT2-R	TTGCCGAGGAGTTTGAGATAAT		
Blimp1-F	AACCTGGCTGCGTGTCAGAAC	182	40
Blimp1-R	CTCGGTTGCTTTAGACTGCTCTG		
PKG1-F	GAGTTGGAGGTTTCGGACGAGT	146	40
PKG1-R	GATGTGCTCCTGCTGTCTTGTG		
FZD1-F	GGCCTGAAGATATGGAGTG	172	40
FZD1-R	GGGGGAAGAAAGTAGGTTGC		
CTNNB1-F	CTCAGTCCTTCACTCAAGAA	102	40
CTNNB1-R	CATCTAATGTCTCAGGGAACA		
TGFB1-F	AATTGAGGGCTTTCGCCTTAG	84	40
TGFB1-R	CCGGTAGTGAACCCGTTGAT		
LRP5-F	GACCTGATGGGACTCAAAGC	156	40
LRP5-R	TCCAGTAGATGTGGTFGTTGG		
IFIT3-F	GGAAACTACGCCTGGGTC	180	40
IFIT3-R	CACCTTCGCCCTTTCATT		
Vimentin-F	GACCAGCTAACCAACGACAA	150	40
Vimentin-R	GTCAACATCCTGTCTGAAAGAT		
Ncad-F	CGAATGGATGAAAGACCCATCC	174	40
Ncad-R	GGAGCCACTGCCTTCATAGTCAA		
KLF4-F	TCCCATCTTTCTCCACGTTC	262	40
KLF4-R	TCCAGGAGATCGTTGAACTC		
STAT3-F	CCAATGGAATCAGCTACAGC	236	40
STAT3-R	GCTGATAGAGAACATTCGACTC		
p21-F	TGGAGACTCTCAGGGTCGAAA	65	40
p21-R	GGCGTTTGGAGTGGTAGAAATC		
p27-F	GAGGGCAGATACGAGTGGCAG	62	40
p27-R	CTGGACACTGCTCCGCTAACC		
cyclinD1-F	CTAGCAAGCTGCCGAACC	90	40
cyclinD1-R	TCCGAGCACAGGATGACC		
β-actin-F	TCCTGTGGCATCCACGAAACT	315	40
β-actin-R	GAAGCATTTGCGGTGGACGAT		

F, forward; R, reverse.

### 2.6 Proliferation assay

Cells from different groups in the 6-well plates were cultured for two weeks respectively. Cell proliferation was determined by CCK8 and colony formation assay as described before [16]. CCK8 assay: experimental and vector cells were plated into 96 well plate with 1500 cells per well. Each group is equipped with 5 wells. After the cells were fully adherent to the wall (0 h), we detected the absorbance values of each group at 24h, 48h, and 72h, accordingly. Colony formation assay: the experimental and vector cells were seeded in a six well plate (1000 cells per well) and cultured for approximately two weeks to form visible colonies. Then we collected, fixed, stained, and counted the clone cells. All experiments were independently repeated three times.

### 2.7 Mobility assay

Cell mobility was detected by conducting wound healing assays. U87, LN-18 and A172 cells with stable ADCY5 expression were seeded in 6-well plates until cells reached at least 90–95 % confluence. The cell layers were carefully creating wounded with pipette tips, and cell migration distance was measured every 12h through phase contrast microscopy.

Transwell assay was done as described before [17]. 700 μl medium of different groups (1 × 10^5^ cells) and 700 μl blank medium (10 % FBS) were added into the upper and lower chambers respectively. The migrated cells on lower chamber were fixed in 4 % paraformaldehyde for 20 min, stained with hematoxylin, and counted under upright microscope (five fields per chamber) after 48h incubation. The invaded cells on the upper chamber were undergone the same operations after removed the Matrigel. Migrating cells were counted under microscopy at 400 × magnification and counted in six random fields. Each assay was repeated in three independent experiments.

### 2.8 Functional enrichment analysis

Functional enrichment analysis was performed by using Functional Annotation Tool DAVID Bioinformatics Resources and the functional enrichment analysis tool (FunRich v3.1.3) software, which provided the analysis of GO terms, protein-protein interactions, protein functional domains, disease associations, bio-pathways, sequence general features, homologies, gene functional summaries, gene tissue expressions, pieces of literature, etc [18]. We have chosen Homo sapiens and only terms with P-value <0.01, minimum count of 3, and enrichment factor of >1.5 were considered as significant.

### 2.9 Initially search for gene regulatory networks

Functional regulatory networks for ADCY5 were determined by the STRING and cBioPorta, a web platform of “genebased” visualizations and analyses and it provided information related to cancer study as we had described before [18]. Network type set such as full STRING network (the edges indicate both functional and physical protein associations), which minimum required interaction score at medium confidence (0.400), and max number of interactors: no more than 10 interactors.

### 2.10 Statistical analysis

All the raw data were extracted from above online database and created by using GraphPad software 8.0 (Inc. La Jolla, CA, USA). The mRNA and methylation expression difference in DiseaseMeth database between tumor and normal tissues were analyzed by the un-paired Student's t-test. Spearman's rank correlation coefficient was utilized to discover the strength of a link between two independent sets of data. The significance of survival differences between ADCY5-low and ADCY5-high expression groups were compared by the log-rank test. The univariate and multivariate cox regression approaches were applied to analyze prognostic factors. Correlations were calculated by the Spearman's correlation analysis. For cell culture, three independent biological replicates were examined for each experiment. *P < 0.05, **P < 0.01, ***P < 0.001 were considered statistically significant.

## 3 Results

### 3.1 mRNA expression of ACs in human GBM tissues

**Fig. 1:**
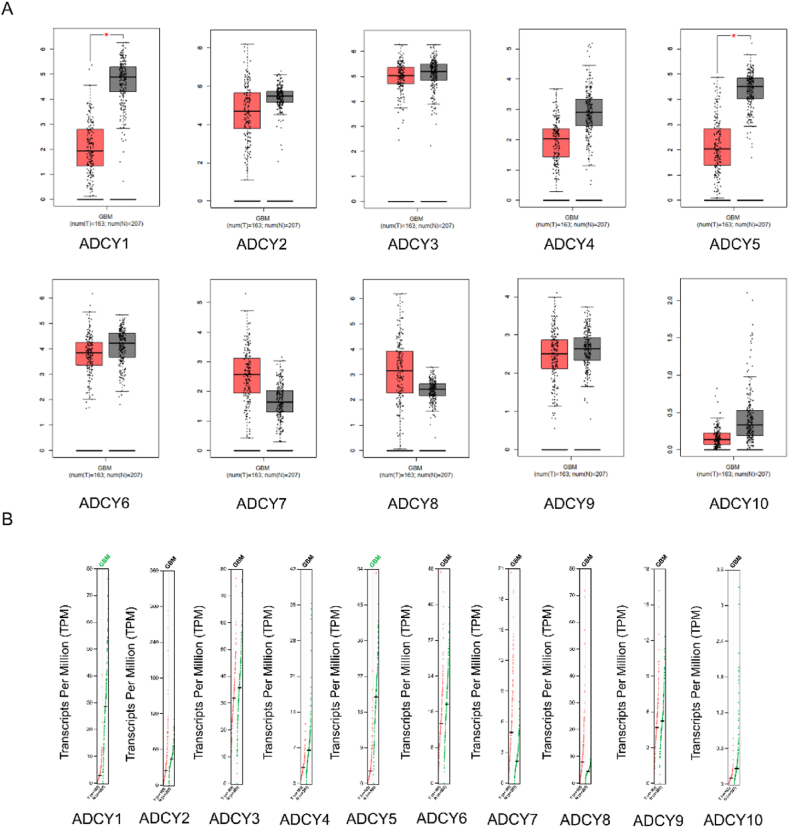
Box plots and gene expression profiles presenting the mRNA levels of ACs in GBM. (A) ACs mRNA expression of GBM, *P < 0.01. (B) Transcripts per million (TPM) of ACs.

To analyze ACs mRNA expression in GBM, we investigated 207 normal brain tissues and 163 GBM tumor tissues in GEPIA online website. As shown in (Fig. 1A and B), ADCY1 and ADCY5 mRNA were down-regulated in GBM comparing to normal tissues with P < 0.01, while these were no significant difference in ADCY2, ADCY3, ADCY4, ADCY6, ADCY7, ADCY8, ADCY9 and ADCY10 mRNA expression.

**Fig. 2:**
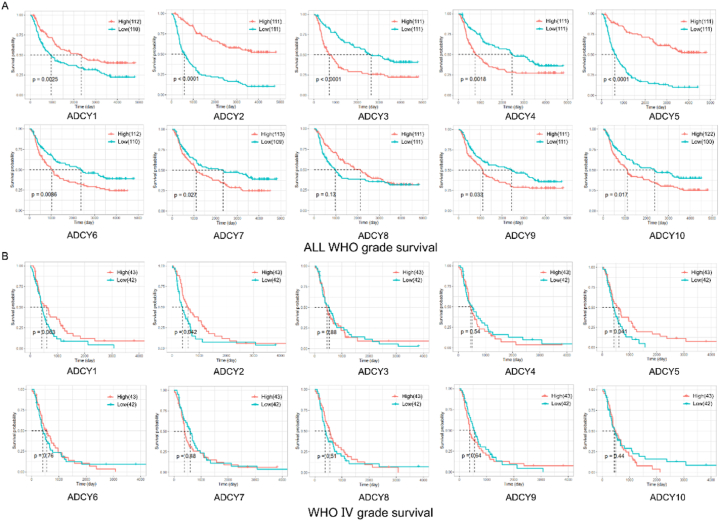
The prognostic value of mRNA level of ACs in glioma. (A) Overall survival: the relationship between ACs expression and prognosis in glioma. (B) Overall survival: prognosis of ACs in GBM.

We further detected the association between ACs and Overall Survival (OS) rates by using GEPIA database. The results revealed that high mRNA expression of ADCY1, ADCY2, and ADCY5 were statistically related to better OS in Glioma patients with P < 0.01, compared with the low expression groups respectively (Fig. 2A). Meanwhile, the survival curves showed that higher level of combined ACs, including ADCY3 (P < 0.0001), ADCY4 (P = 0.0018), ADCY6 (P = 0.0086), ADCY7 (P = 0.027), ADCY8 (P < 0.001), ADCY9 (P = 0.033) and ADCY10 (P = 0.017) predicted poor prognosis in Glioma (Fig. 2A). Then we examined the individual ACs mRNA expression and founded that ADCY2 (P = 0.0042) and ADCY5 (P = 0.0041) were associated with improved OS in GBM (Fig. 2B). These results showed that ACs may be useful for GBM prediction and down-expression of ADCY5 played an important role in GBM.

### 3.2 ADCY5 expression in various tumor types

**Fig. 3:**
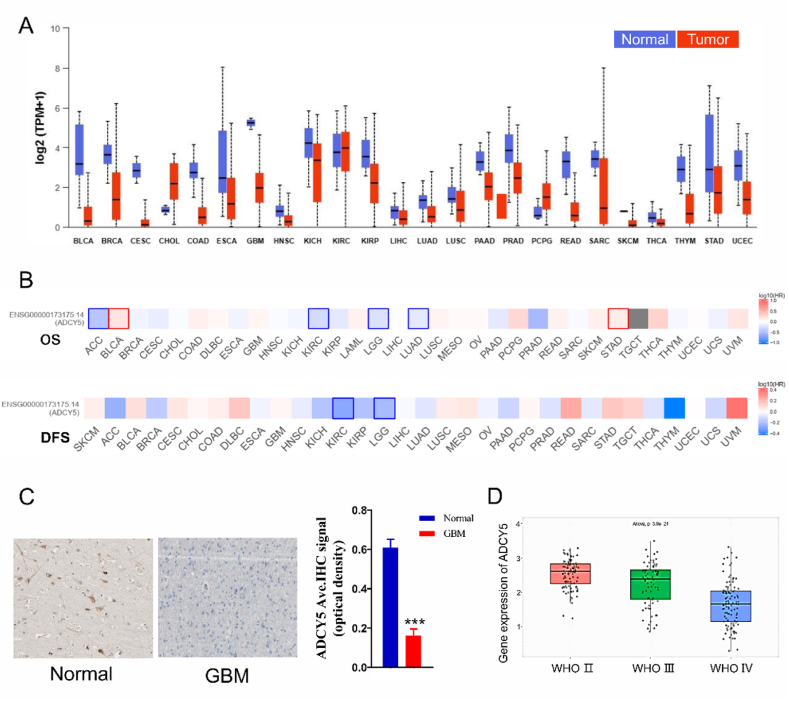
Expression pattern and prognostic value of ADCY5 in GBM. (A) Pan-cancer analysis of ADCY5 in multiple tumors according to TCGA databases. (B) Pan-cancer OS and DFS in multiple tumors expressing ADCY5. (C) Representative IHC analysis showed ADCY5 lower expression in GBM base on HPA database, ***P < 0.001 (D) The higher the grade of glioma, the lower the mRNA expression of ADCY5 base on CGGA databases.

ADCY5 mRNA expression in multiple cancers and their normal tissues were extracted through TCGA, HPA and CGGA databases. Additionally, we found that ADCY5 mRNA was down-regulated in nearly all the tumors including GBA, Esophageal cancer (ESCC), Breast cancer (BRCA), Kidney renal clear cell carcinoma (KIRC) et al. (Fig. 3A). Heat map in survival analysis indicated that there were better OS in high-expressed ADCY5 groups including BRCA, GBM, Stomach adenocarcinoma (STAD) and better individual Disease Free Survival (DFS) individually in Diffuse Large B-cell Lymphoma (DLBC), Cervical squamous cell carcinoma (CESC), GBM groups et al. (Fig. 3B). However, worse OS in Disease Free Survival (DFS) could be seen in lower ADCY5 expressed groups including Adrenocortical carcinoma (ACC), KIRC groups et al. (Fig. 3B).

### 3.3 Clinical significance of ADCY5 expression in GBM

**Fig. 4:**
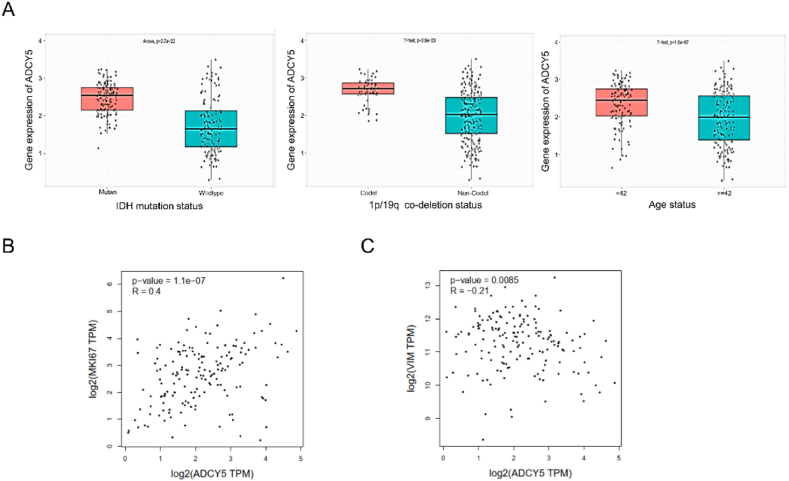
Clinical significance of ADCY5 expression in GBM. (A) ADCY5 expression in GBM patients based on IDH mutation status, and 1p/19q codeletion status, Age status. (B) The collection between ADCY5 and a proliferation marker (Ki-67). (C) The collection between ADCY5 and a invasion marker (Vimentin).

We detected the ADCY5 protein expression in GBM based on HPA database and found that ADCY5 proteins were significantly down-regulated in GBM, comparing with normal brain (Fig. 3C, P < 0.001). ADCY5 mRNA expression was closely related to tumor grade according to CGGA databases, the higher the tumor grade, the lower the expression levels (Fig. 3D, P < 0.001). Meanwhile, statistical significance was observed between high ADCY5 expression and IDH mutation (Fig. 4A, P < 0.001), 1p/19q codeletion status (Fig. 4A, P < 0.001), age (Fig. 4A, P < 0.001). ADCY5 expression was negatively related to proliferation marker MKi67 (Fig. 4B, P < 0.001) and invasion markers VIM (Fig. 4C, P < 0.001), implying ADCY5 may influence cancer cell proliferation and invasion.

CGGA databases with ADCY5 mRNA expression values and clinical information were further screened out. The correlation between ADCY5 mRNA expression and multiple clinical characteristics were studied. We found that high-expressed ADCY5 was significantly associated with ages, grade, IDH mutation, 1p19q_codeletion, radiotherapy and chemotherapy (Table 3, P < 0.05). But there were no important expression relationship between ADCY5 mRNA and gender, PRS types (Table 3, P > 0.05). Univariate analysis showed that prognosis of GBM was correlated with ADCY5 expression, age, grade, IDH mutation, 1p19q_codeletion, radiotherapy and chemotherapy (Table 4, P < 0.05). While Cox regression analysis suggested that high-expressed ADCY5, grade, IDH mutation, 1p19q_codeletion, radiotherapy, and chemotherapy were independent prognostic factors in GBM (Table 4, P < 0.05). Meanwhile, ADCY5 methylation expression and related clinical characteristics based on CGGA databases were also investigated. There was only clinical significance between ADCY5 expression and tumor grade (Table 5, P < 0.05), and further analysis is needed for large samples. These studies implied that ADCY5 may be an important biomarker for the occurrence, development and prognosis in GBM.

**Table 3:** Correlation between ADCY5 expression and different clinical factors based on CGGA.

Clinical pathology	N	ADCY5 mRNA		χ2	P
		low expression (182)	high expression (139)		
**Gender**				0.316	0.24
Male	199	116	83		
Female	122	66	56		
**Year**				24.668	<0.001
<42	149	70	79		
≥42	172	112	60		
**Clinical stage**				32.264	<0.001
WHO II	103	33	70		
WHO III	79	36	43		
WHO IV	139	113	26		
**IDH_mutation_status**				42.722	<0.001
Wildtype	146	113	33		
Mutant	175	69	106		
**1p19q_codeletion_status**					
Non-codel	254	171	83	28.435	<0.001
Codel	67	11	56		
**Radio status**					
No	51	31	20	6.279	0.0268
Yes	270	151	119		
**Chemo status**					
No	62	35	27	5.662	0.0323
Yes	259	147	112		
**PRS type**				0.264	0.63
Primary	231	132	99		
Recurrent	60	32	28		
Secondary	30	18	12

**Table 4:** Univariate and Multivariate analysis of prognosis in GBM.

Variable	Univariate analysis		Multivariate analysis	
	HR (95 % CI)	P	HR (95 % CI)	P
ADCY5 mRNA (High vs Low)	0.507(0.312, 1.742)	2.12 × 10^−4^	0.884(0.703, 2.673)	1.7 × 10^−4^
Age (≥42 vs < 42)	0.662(0.528, 1.372)	4.56 × 10^−3^	0.727(0.453, 2.027)	0.326
Gender (female vs male)	1.662(1.602, 4.872)	0.782	–	–
Grade (high vs low)	0.477(0.188, 1.78)	4.27 × 10-4	0.621(0.308, 2.228)	3.74 × 10-3
IDH_mutation (yes vs no)	0.581(0.242, 1.004)	5.52 × 10-3	0.482(0.476, 1.227)	4.77 × 10-3
1p19q_codeletion (yes vs no)	0.306(0.325, 0.846)	8.32 × 10-5	0.722(0.472, 1.565)	5.52 × 10-5
Radio (yes vs no)	0.421(0.179, 1.021)	4.28 × 10-4	0.586(0.334, 1.562)	5.26 × 10-3
Chemo (yes vs no)	0.473(0.532, 2.172)	2.24 × 10-3	0.376(0.428, 1.872)	3.2 × 10-3

**Table 5:** Correlation between ADCY5 methylation and different clinical factors based on CGGA.

Clinical pathology	N	ADCY5 mRNA		χ2	P
		low expression (74)	high expression (77)		
**Gender**				0.269	0.76
Male	89	43	46		
Female	62	31	31		
**Year**				0.273	0.67
<42	69	39	30		
≥42	72	35	37		
**Clinical stage**				8.528	0.013
WHO II	61	25	36		
WHO III	47	23	24		
WHO IV	41	26	17		
**Histology**				0.359	0.44
AA, AG, AOA	24	14	10		
A, AO, O, rAO, rA	86	35	51		
GBM	31	19	12		
rGBM, sGBM	10	6	4		
**Censor**					
NA	11	3	8	0.481	0.671
0	50	19	31		
1	90	52	38		
**OS**					
NA	11	3	8	0.632	0.827
Low	83	50	33		
High	57	21	36		

### 3.4 Methylation of the ADCY5 promoter in GBM

**Fig. 5:**
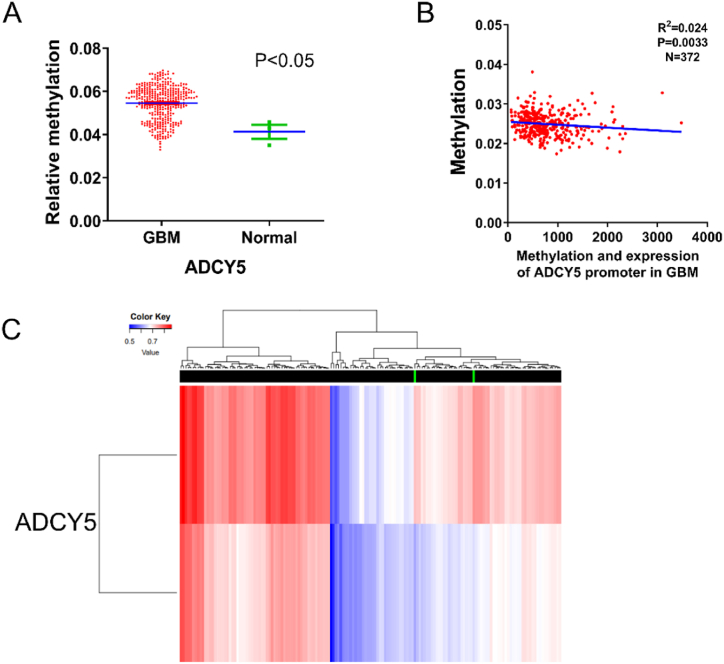
The epigenetic alterations on mRNA expression of ADCY5 in GBM. (A) The methylation levels of ADCY5 gene promoter in GBM and normal samples were analyzed by MethHC database. (B) The relationship between expression of methylation and expression of ADCY5 in GBM according to MethHC database. (C) The methylation status of ADCY5 in GBM by DiseaseMeth dataset.

Abnormal DNA methylation played important role in the occurrence and development of GBM [19,20], Meanwhile, abnormal promoter methylation contributes a key mechanism for the silencing of tumor suppress genes in carcinogenesis [21]. To verify whether the downregulated of ADCY5 mRNA was related to its methylation, we concurrently analyzed the promoter methylation of ADCY5 in GBM through analyzing online MethHC and DiseaseMeth dataset. Results from MethHC dataset showed that ADCY5 was hypermethylated in 372 GBM tissues compared with normal tissues with P < 0.05 (Fig. 5A) and high expressed ADCY5 was negatively correlated with the promoter CpG methylation (Fig. 5B, P = 0.0033). Meanwhile, hypermethylation of the ADCY5 promoter was still seen in GBM in DiseaseMeth dataset, which was the same as previous results (Fig. 5C).

### 3.5 ADCY5 mRNA downexpressed in GBM cells and over-expression of ADCY5 suppresses cancer cell growth and viability of GBM cells

**Fig. 6:**
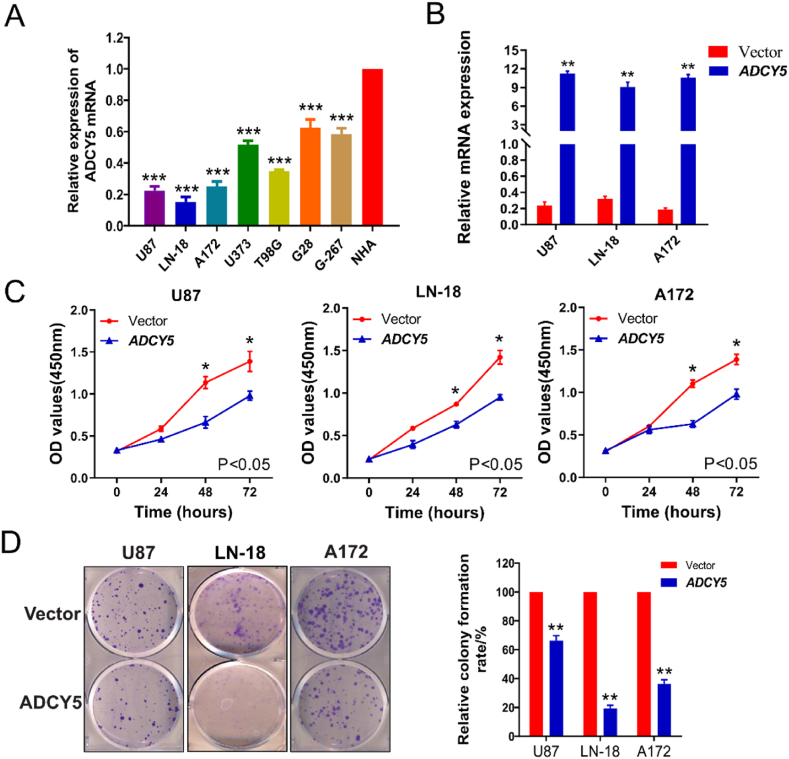
Cell function of ADCY5 in GBM cells. (A) ADCY5 expression was detected by qRT-PCR in GBM cell lines and Human Astrocyte NHA, ***P < 0.001. (B) Ectopic expression of ADCY5 in GBM cell lines was measured by qRT-PCR, **P < 0.01. (C) CCK8 assay of GMB cells demonstrated that ADCY5 suppressed cell vibility, *P < 0.05. (D) Colony formation assay showed that re-expressed ADCY5 expression in GBM groups inhibited the formation of cell clone, **P < 0.01.

Next, we examined the tumor-related function of ADCY5 in GBM as it was responsible for the endogenous downregulation. Firstly, we detected the ADCY5 mRNA expression in seven GBM cell lines by qRT-PCR and found that ADCY5 expression was down-regulated in all the GBM cell lines (Fig. 6A, P < 0.001). To assess the tumor suppressive functions, we further transfected pcDNA3.1(+)-ADCY5 expressed plasmids into U87, LN-18 and A172 cell lines. Re-expression of ADCY5 after stable transfection was detected by qRT-PCR (Fig. 6B, P < 0.01). To ascertain whether ADCY5 is a functional TSG in GBM, we applied CCK-8 and colony formation assays to detect cell viability and proliferation after over expression of ADCY5 and empty vector in U87, LN-18 and A172 cell lines. CCK-8 assays showed over-expression of ADCY5 could suppress cell proliferation (Fig. 6C, P < 0.05). Colony growth assays indicated re-expression of ADCY5 could decrease colony formation of these GBM cells (Fig. 6D, P < 0.01).

### 3.6 ADCY5 inhibits cell migration and invasion in vitro

**Fig. 7:**
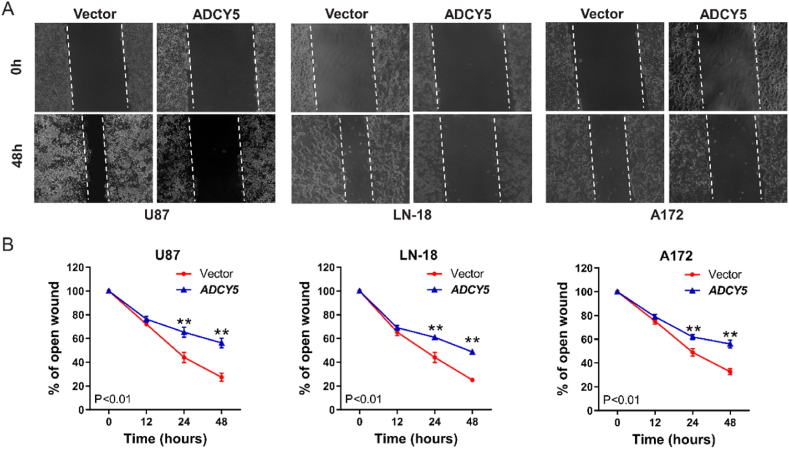
ADCY5 suppressed cell migration. (A) Wound-healing assays showed overexpression of ADCY5 suppressed cell migration. (B) The inhibitory effect of U87, LN-18 and A172 cells were decreased by re-expressed ADCY5 expression, ***p < 0.01.

**Fig. 8:**
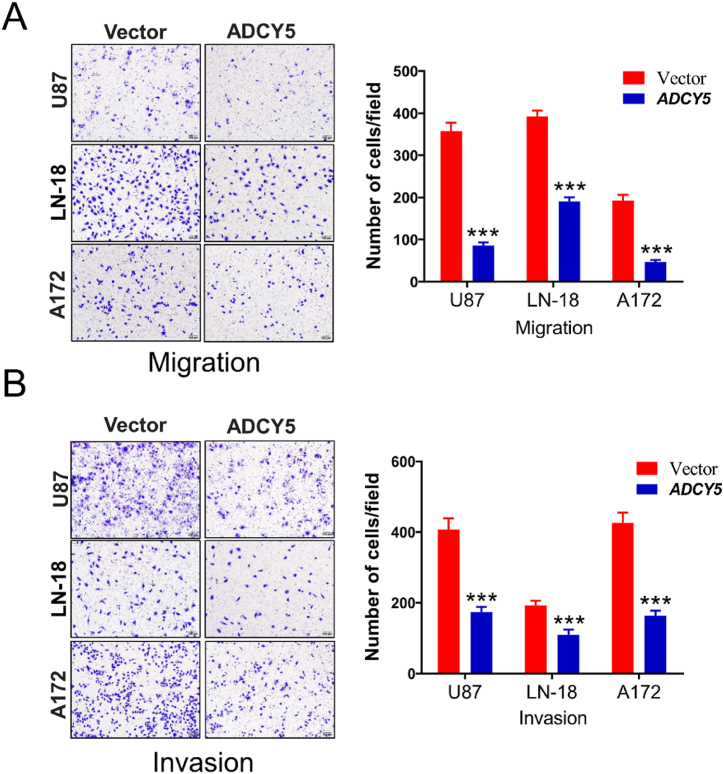
ADCY5 inhibited cell migration/invation. (A) Transwell assay showed that the number of migration GBM cells in ADCY5 over-expressed groups were lower than that in control group, ***P < 0.001. (B) The inhibitory invasion effects were decreased by ADCY5 over-expressed cells, ***P < 0.001.

An important hallmark of GBM cells is its ability to invade surrounding tissues and metastasize to brain [3]. We further examined if ADCY5 affected migratory and invasive properties of GBM cells in vitro. Wound-healing scratch assays showed that compared with empty vector, ectopic expression of ADCY5 in cell lines delayed the closure of wound gaps as observed at 24 h and 48 (Fig. 7A and B, P < 0.01). Transwell assays demonstrated U87, LN-18 and A172 cells stably expressed ADCY5 could inhibit migration and invasion without or with Matrigel (Fig. 8A and B, P < 0.001).

**Fig. 9:**
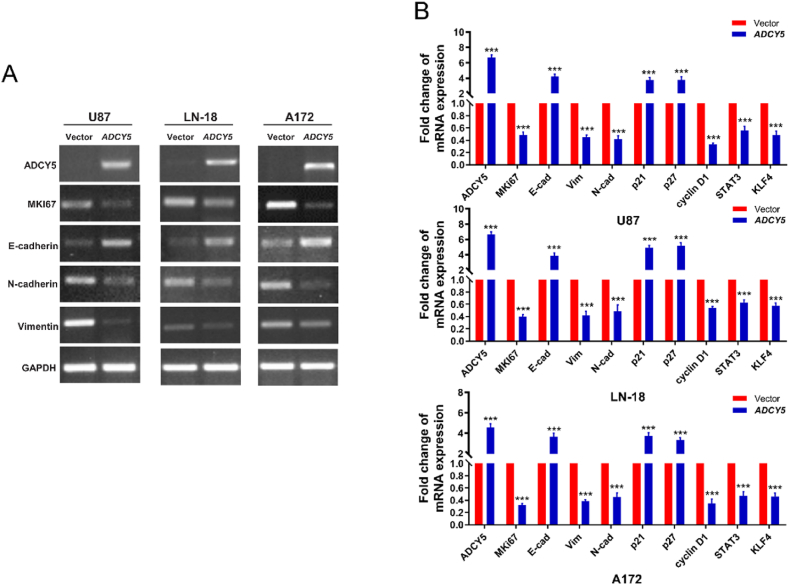
ADCY5 inhibited cell proliferation and EMT. (A) RT-PCR showed representative GBM cell proliferation and EMT relative markers in ADCY5 over-expressed U87, LN-18 and A172 cells. *Indicates significantly downregulated bands. (B) ADCY5 restored relative mRNA expression of proliferation, EMT markers and its down-stream stem cell markers, ***P < 0.001.

We further investigated the underlying mechanism of ADCY5 on regulating of cell growth, viability, migration and invasion. RT-PCR and qRT-PCR showed mRNA expression of cell proliferation-related MKi67 and the mesenchymal markers Vimentin and N-cadherin decreased, while the epithelial markers E-cadherin increased (Fig. 9A and B, P < 0.001). In addition, the expression of cell proliferation-related upstream p21 and p27 were increased while cyclinD1 was decreased (Fig. 9B, P < 0.001). Considering the intimate connection between epithelia-mesenchymal transition (EMT) and cancer stemness, we also found the expression of STAT3 and KLF4 were decreased in ADCY5 overexpressing cells (Fig. 9B, P < 0.001), suggesting ADCY5 was capable of inhibiting stemness of tumor cells.

### 3.7 Genetic alteration, co-expression and interaction analyses of ADCY5 in GBM

**Fig. 10:**
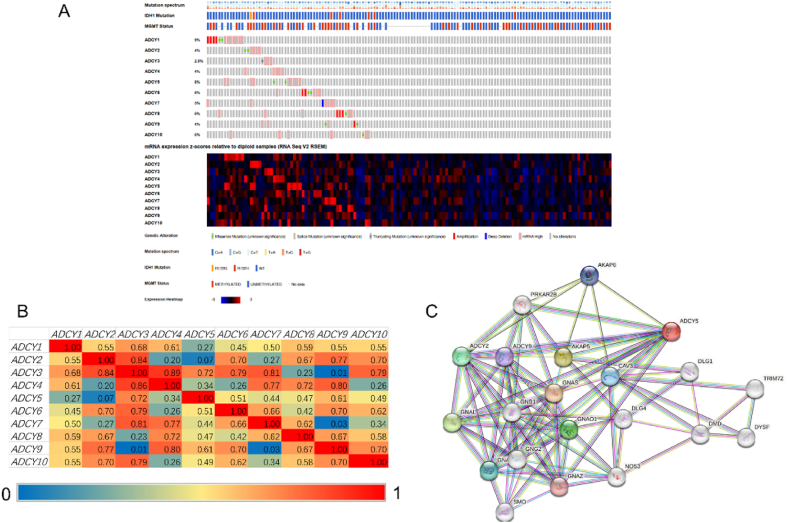
Relative levels of ADCY5 and mutation analysis in GBM (cBioPortal). (A) Summary of alterations and mutation in AC isoforms. (B) Correlation analysis of AC isoforms by heatmap. (C) Gene–gene interaction network among AC isoforms in the cBioPortal database.

To examine the possible regulatory mechanisms, we analyzed ADCY5 genetic alteration, correlation, and network simultaneously. The data obtained from the TCGA database (PanCancer Altas) was applied in the research. Results showed that the proportion of genetic alterations in ACs ranged from 2.8 to 9 % respectively (ADCY1, 9 %; ADCY2, 3 %; ADCY3, 2.8 %; ADCY4, 4 %; ADCY5, 8 %; ADCY6, 6 %; ADCY7, 5 %; ADCY8, 6 %; ADCY9, 4 %; ADCY10, 6 % (Fig. 10A). Moreover we detected the mRNA expression correlation between each Acs members via utilizing the cBioPortal online tool, and found statistical significance as bellows: ADCY1 with ADCY2, ADCY3, ADCY4, ADCY8, ADCY9 and ADCY10; ADCY2 with ADCY1, ADCY3, ADCY6, ADCY8, ADCY9 and ADCY10; ADCY3 with ADCY1, ADCY2, ADCY4, ADCY6, ADCY7 and ADCY10; ADCY4 with ADCY1, ADCY3, ADCY7, ADCY8 and ADCY10; ADCY5 with ADCY3, ADCY6 and ADCY10; ADCY6 with ADCY2, ADCY3, ADCY5, ADCY6, ADCY7, ADCY9 and ADCY10; ADCY7 with ADCY3, ADCY4, ADCY6, ADCY7 and ADCY8; ADCY8 with ADCY1, ADCY2, ADCY4, ADCY7, ADCY9 and ADCY10; ADCY9 with ADCY1, ADCY2, ADCY4, ADCY5, ADCY6, ADCY8 and ADCY10; ADCY10 with ADCY1, ADCY2, ADCY3, ADCY6, ADCY8 and ADCY9 (Fig. 10B). STRING network analysis showed that cAMP regulated genes including AKAP6 and AKAP5, Wnts up-stream genes including GNAS, GNB1 and GNAL were closely associated with ADCY5 (Fig. 10C).

### 3.8 Potential biomolecular networks associated with ADCY5

**Fig. 11:**
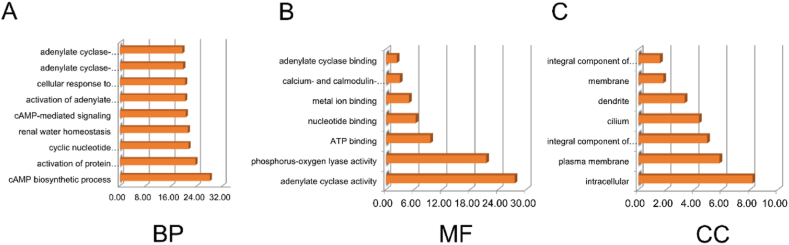
The enrichment analysis of ADCY5 in GBM. (A) Biological processes. (B) Biological process. (C) Cellular components.

Molecular function (MF), biological process (BP), and cellular components (CC) were used to detect the possible gene function of ADCY5. We found that the biological processes involved in these genes mainly included biological regulation, metabolic processes, as well as cell proliferation and growth (Fig. 11A). Classification of cellular components mainly concentrated in cell membranes, nuclei et al. (Fig. 11B). The molecular functions mainly included protein binding, ion binding, nucleic acid binding transcription factor activity, and other functions (Fig. 11C).

**Fig. 12:**
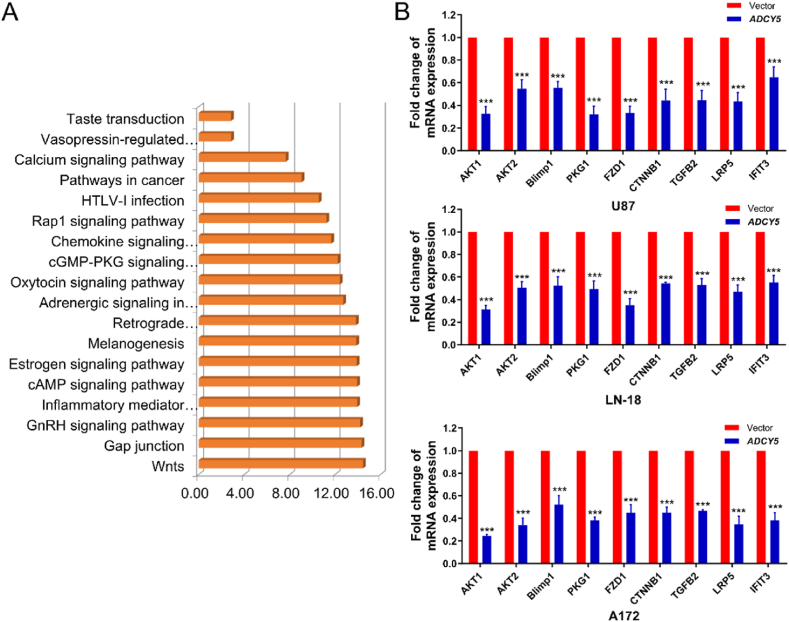
ADCY5 influenced biomolecular network. (A) Clusters from KEGG pathway enrichment analysis of ADCY5-related genes predicted with the list ontology sources. (B) ADCY5 inhibited oncogenic signaling and its down-stream EMT related stem cell markers, ***P < 0.001.

KEGG pathway enrichment analysis was used to detect latent regulatory mechanisms of ADCY5 and we found several cancer-related pathways participated in its carcinogenesis and progression including pathways in cancer, cAMP pathway, cGMP-PKG pathway, Wnts pathway, etc. (Fig. 12A). To obtain better comprehensive knowledge of the underlying molecular mechanisms in which ADCY5 exerted its anti-cancer ability, we searched for differentially expressed target genes of ADCY5 by RNA sequencing analysis. Down-regulated gene pathways were mainly involved in cell proliferation, metabolism, invasion. and particularly alliterated in different pathways in cancer pathway including cAMP/AKT pathway, cGMP-PKG pathway, Wnts pathway contributed to dramatic differences in expression profiles in GMB cells (Fig. 12B). These data indicated the regulation of main oncogenic signaling pathways by ADCY5 in GBM progression.

## 4 Discussion

Malignant glioma is the most common primary brain tumor, in which GBM is the most malignant and highly invasive Glioma, with poor therapeutic effect and high mortality. The main reason for poor prognosis in GBM is due to lack of effective early diagnosis indicator and intervention method, while the special tumor microenvironment in the brain and the unique genetic heterogeneity of Glioma are also considered as important factors for poor treatment [22,23]. Previous study had showed that GBM cells could mimic the molecular circuits and the characteristics of myeloid cells to evade immune surveillance, thus achieving immune escape. Immunological indicators, tumor stage and other indicators are insufficient to evaluate the occurrence and prognosis of GBM patients [24]. Therefore, further study of GBM pathogenesis and screening of the predictable molecular targets would have a positive significance for improving the prognosis of GBM and realizing individualized treatment.

Previous research on ACs mainly focused on glucose metabolism, epilepsy, movement disorders and related diseases [25]. At present there are few reports on ACs in human malignant tumors [[10], [11], [12]]. In this research, we revealed the integrated study which systematically profiled mRNA expression, genetic and epigenetic alteration, prognostic value and regulatory network for ADCY5 and its family members in GBM for the first time. We identified ADCY5 as a novel tumor suppressor in GBM and its capacity of inhibiting their growth and invasion. Moreover, we discovered that ADCY5 functioned by targeting multiple oncogenic signaling pathways.

ADCY5 was located on chromosome 3q21.1, with a total length of about 167 kb, encoded Adenylyl cyclase V-type protein ADCY5 [26]. The ADCY5 coding protein consisted of 1261 amino acid residues and had a total of four domains: two 6-helical cross-sectional transmembrane domains M1 and M2 and two cytoplasmic catalytic domains C1 and C2. When C1 and C2 combined, a catalytic bag would be formed to convert ATP into cAMP [27]. ADCY5 was mostly expressed in the heart, brain, and pancreas, and catalyzed the production of cAMP, as cAMP itself acted an important regulatory factor in glucose metabolism, lipid metabolism and carcinogenesis [28].

ACs were found to participate in several cancer types as they could regulate various cellular functions including cell cycle progression [29]. In this study, ADCY1 mRNA, ADCY5 mRNA and protein were down-regulated in GMB compared with normal brain tissues via bioinformatics analysis. The prognostic value of ACs in GBM were also acquired using GEPIA database and results showed that higher expression of ADCY1, ADCY2, and ADCY5 were associated with better OS rates, and over-expressed ADCY2 and ADCY5 were related to improved OS rates in GBM.

Furthermore, TCGA database indicated that ADCY5 was downregulated in nearly all cancer types, we thus investigated the clinical significance of ADCY5 mRNA expression in GBM by downloading corresponding clinical data in CGGA database. Results showed that there were no statistically significant difference in ADCY5 mRNA expression between gender and PRS types. While it was found that there were significant differences in ADCY5 mRNA expression in the aspects of age, grade, IDH mutation, 1p19q_codeletion, radiotherapy and chemotherapy groups. Subsequently we conducted Cox survival regression verification, univariate analysis and multivariate analysis, which showed that ADCY5 mRNA expression and grade, IDH mutation, 1p19q_codeletion, radiotherapy and chemotherapy were independent prognostic factors. Thus, this was the first report that ADCY5 acted as an independent prognostic factor in GBM, indicating that the potential tumor-suppressive role of ADCY5 in GBM.

Abnormal expression of epigenetic markers causes the structural changes in the three-dimensional conformation of chromatin, interfering with gene expression to some extent and thus leads to carcinogenesis [30,31]. This study detected the methylation of ADCY5 in multiple GBM tissues, and found that ADCY5 would highly methylated in GBM and negatively correlated with mRNA expression, suggesting that ADCY5 gene methylation was ubiquitous in GBM. Moreover, our present findings demonstrated the important roles for ADCY5 in malignant progression and metastasis of GBM. Our data also revealed that ectopic expression of ADCY5 could suppress cell growth and viability by regulating cell proliferation related gene transcription such as p21, p27 and cyclin D1. Meanwhile ADCY5 could also suppress cell migration and invasion by interfering with EMT and its downstream stemness genes. Thus, our data revealed that ADCY5 inhibited cell viability, proliferation, migration/invasion, indicating that ADCT5 was a tumor suppress gene in GBM.

In order to investigate how ADCY5 functioned, biologically functional enrichment analysis was performed by GO and KEGG pathway enrichment analyses. We found the significant relevance between ADCY5 and the downstream signaling pathways, such as cAMP/AKT pathway, cGMP-PKG pathway, Wnts pathway. As the published research revealed that dysregulation of these pathways were related to the pathogenesis of various diseases, the extra-activation of ACs, along with the related upstream and downstream regulators, were considered as principal underlying targets of novel anticancer therapies and markers of carcinogenesis. The occurrence, progression, metastasis, and drug resistance of major cancers were regulated through the cAMP signal downstream of β-adrenergic receptors (β-ARs), which were coupled to stimulatory G-protein (Gs) [32,33]. In this research, ADCY5 combined with protein-coupled receptors and the downstream cAMP signaling pathway. Additionally, cAMP played a key role in endoskeleton remodeling, cell proliferation, adhesion and EMT by preventing AKT, PKG and Wnts activation, thus leading to the inhibition of EMT consequently [34,35]. We also found that ADCY5 antagonized downstream AKT, PKG and Wnts pathways in GMB cells, which may provide newly insight into the discovery of possible molecular mechanisms and encourage further consideration in GBM.

In summary, the research suggested that ADCY5 mRNA would be decreased in GBM due to DNA promoter hypermethylation and gene mutation. Furthermore, significant relationship between ADCY5 and clinical outcomes were also detected and the data showed that ADCY5 may provide valuable information to the clinical oncologist in GBM. However, there were limitations in this research. First, some of the bioinformatics tools used had limited functionality and clinical samples, which required further confirmation at different database. Second, there was no enough evidence to confirm the direct biological function in vivo and molecular mechanism of ADCY5 in GBM. In the future, we intend to deeply evaluate the value of ADCY5 as a potential biomarker for GBM. Taking together, these results implied ADCY5 may have prognostic value and act as tumor suppress genes in GBM.

## Data availability statement

All data generated in this study are available from the corresponding author upon reasonable request.

## Funding

This study was supported by Natural Science Foundation of Chongqing of China (cstc2020-jcyj-msxmX0355), Chongqing Science and Health Medical Research Project (2024MSXM055).

## Availability of data and materials

The datasets generated and/or analyzed during the current study are available in the GEPIA database (http://gepia.cancer-pku.cn/), the human protein atlas (http://www.proteinatlas.org/) Kaplan-Meier database (http://kmplot.com/analysis/), STRING (https://string-db.org/), DAVID (https://david.ncifcrf.gov/) and cBioPorta (http://www.cbioportal.org/).

## Ethics approval and consent to participate

Not applicable.

## Consent for publication

Not applicable.

## CRediT authorship contribution statement

**Wang Can:** Writing – original draft, Methodology, Data curation. **Wen Yan:** Project administration, Data curation, Conceptualization. **Huang Luo:** Data curation, Conceptualization. **Zhang Xin:** Investigation. **Luo Yan:** Formal analysis. **Liu Deqing:** Methodology. **Tu Honglei:** Supervision. **Li Xiaoyu:** Resources, Project administration. **Sui Jiangdong:** Visualization, Funding acquisition. **Xie Yue:** Project administration. **Li Jing:** Writing – review & editing, Writing – original draft, Project administration, Funding acquisition.

## Declaration of competing interest

The authors declare that they have no known competing financial interests or personal relationships that could have appeared to influence the work reported in this paper.
