# Supplementary figures and images for: ADCY5 act as a putative tumor suppressor in glioblastoma: An integrated analysis
Source: Heliyon. 2024 Aug 27;10(17):e37012. doi: 10.1016/j.heliyon.2024.e37012 (PMC11419897; doi:10.1016/j.heliyon.2024.e37012)

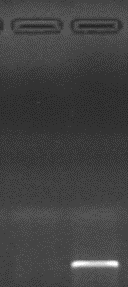

Supplement: Multimedia component 1 [file mmc1.zip › Uncropped gels/A172-ADCY5.png]

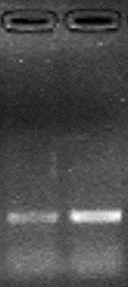

Supplement: Multimedia component 1 [file mmc1.zip › Uncropped gels/A172-E-cadherin.png]

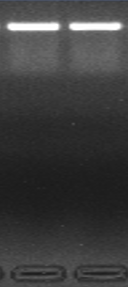

Supplement: Multimedia component 1 [file mmc1.zip › Uncropped gels/A172-GAPDH.png]

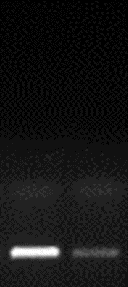

Supplement: Multimedia component 1 [file mmc1.zip › Uncropped gels/A172-MKI67.png]

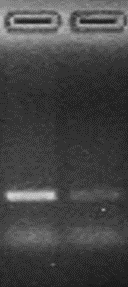

Supplement: Multimedia component 1 [file mmc1.zip › Uncropped gels/A172-N-cadherin.png]

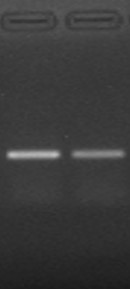

Supplement: Multimedia component 1 [file mmc1.zip › Uncropped gels/A172-Vimentin.jpg]

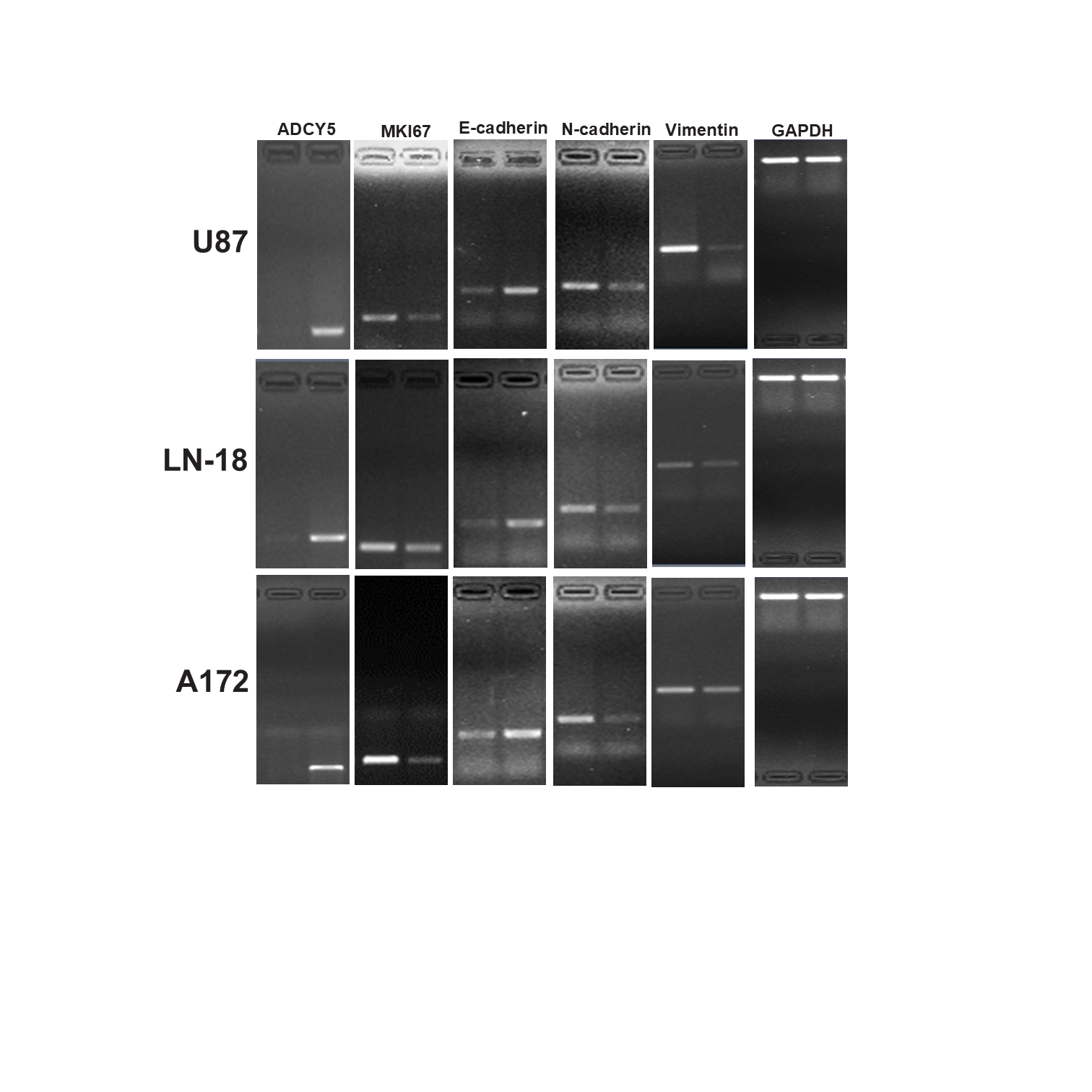

Supplement: Multimedia component 1 [file mmc1.zip › Uncropped gels/Fig9A.tif]

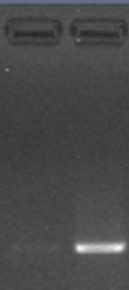

Supplement: Multimedia component 1 [file mmc1.zip › Uncropped gels/LN-18-ADCY5.jpg]

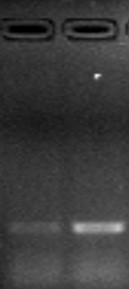

Supplement: Multimedia component 1 [file mmc1.zip › Uncropped gels/LN-18-E-cadherin.jpg]

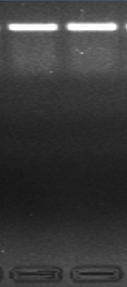

Supplement: Multimedia component 1 [file mmc1.zip › Uncropped gels/LN-18-GAPDH.png]

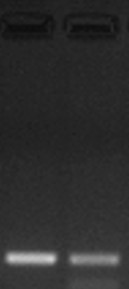

Supplement: Multimedia component 1 [file mmc1.zip › Uncropped gels/LN-18-MKI67.jpg]

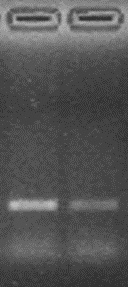

Supplement: Multimedia component 1 [file mmc1.zip › Uncropped gels/LN-18-N-cadherin.jpg.png]

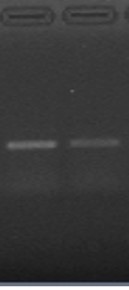

Supplement: Multimedia component 1 [file mmc1.zip › Uncropped gels/LN-18-Vimentin.jpg]

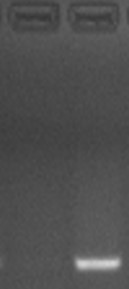

Supplement: Multimedia component 1 [file mmc1.zip › Uncropped gels/U87-ADCY5.jpg]

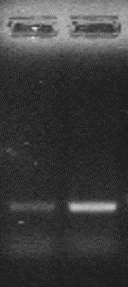

Supplement: Multimedia component 1 [file mmc1.zip › Uncropped gels/U87-E-cadherin.png]

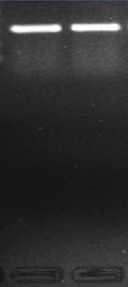

Supplement: Multimedia component 1 [file mmc1.zip › Uncropped gels/U87-GAPDH.png]

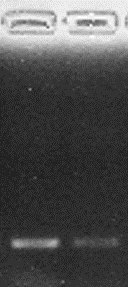

Supplement: Multimedia component 1 [file mmc1.zip › Uncropped gels/U87-MKI67.png]

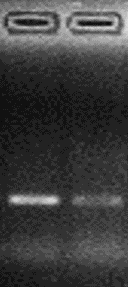

Supplement: Multimedia component 1 [file mmc1.zip › Uncropped gels/U87-N-cadherin.png]

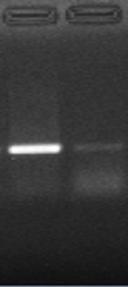

Supplement: Multimedia component 1 [file mmc1.zip › Uncropped gels/U87-Vimentin.png]
